# Is polycystic ovary syndrome appropriately diagnosed by obstetricians and gynaecologists across China: a nationwide survey

**DOI:** 10.1186/s13048-021-00780-6

**Published:** 2021-02-03

**Authors:** Deng Yan, Wang Yan-Fang, Zhu Shi-Yang, Ma Rui-Lin, Ding Xue-Song, Ma Xiao, Xue Wei, Sun Aijun

**Affiliations:** Department of Obstetrics and Gynecology, Peking Union Medical College, Peking Union Medical College Hospital, Chinese Academy of Medical Sciences, No.1 Shuaifuyuan, Dongcheng District, 100730 Beijing, China

**Keywords:** Polycystic ovary syndrome, Diagnosis, Obstetricians and gynaecologists, Survey

## Abstract

**Background:**

To describe the diagnostic criteria used and their application accuracy in the practice of polycystic ovary syndrome (PCOS) caring among obstetricians and gynaecologists across China.

**Methods:**

This was an Online cross-sectional survey of Obstetricians and gynecologists involved in PCOS caring conducted via the largest continuing education platform of obstetrics and gynecology across China from September 2019 to November 2019.

**Results:**

A total of 2,328 respondents were eligible for the final analysis. Of these, 94.5 % were general obstetricians and gynaecologists (Ge-ObGyn), and 5.5 % were reproductive endocrinologists (Re-ObGyn). Overall, the most frequently used criteria were the Androgen Excess and Polycystic Ovary Syndrome Society (AE-PCOS) criteria (48.2 %), followed by the Rotterdam criteria (35.7 %) and NIH criteria (12.1 %). Of the respondents, 31.3 % used their diagnostic criteria in their clinical practice. More respondents who chose the Rotterdam criteria could accurately apply the diagnostic criteria than those who chose the AE-PCOS criteria (41.2 % vs. 32.1 %, *P* < 0.001). Compared with Ge-ObGyn, Re-ObGyn were less likely to use the AE-PCOS criteria (adjusted odds ratio, 0.513; 95 % CI, 0.328–0.802; *P* < 0.05) and 1.492 times more likely to accurately use their criteria (95 % CI, 1.014–2.196; *P* < 0.05).

**Conclusions:**

Less than one-third of obstetricians and gynaecologists across China could accurately use the diagnostic criteria they choose to diagnose PCOS. There is an urgent need to train obstetricians and gynaecologists on PCOS diagnosis in an effort to improve the medical care quality of patients with PCOS.

## Introduction

Polycystic ovary syndrome (PCOS) is the most common gynaecological endocrine disease in women of reproductive age. The clinical manifestations of PCOS include various degrees of reproductive dysfunction and endocrine and metabolic abnormalities[1]. The incidence of PCOS varies in reports, ranging from 6–20 %[2], which is highly related to the diagnostic criteria used in the investigations. Currently, the top three most widely used diagnostic criteria for PCOS are the Rotterdam criteria, NIH criteria, and Androgen Excess and Polycystic Ovary Syndrome Society (AE-PCOS) criteria. The 2003 Rotterdam criteria are the most widely used criteria worldwide, and their definition of PCOS is as follows: (1) oligo-ovulation or anovulation, (2) clinical or biochemical hyperandrogenism, and (3) polycystic ovary (PCO) on ultrasound examination; the diagnosis is made when two out of the aforementioned three items were fulfilled and other diseases were excluded[3]. The 1990 NIH criteria did not include the controversial PCO on ultrasound examination item, and it was necessary to have both oligo-ovulation or anovulation and hyperandrogenism and exclude other diseases[4]. Therefore, the NIH criteria are stricter, and the Rotterdam criteria cover a wider range of patients. The 2006 AE-PCOS criteria define PCOS as follows: hyperandrogenism is a prerequisite, with either oligo-ovulation/anovulation or hyperandrogenism, and exclude other diseases[5]. The AE-PCOS criteria cover a wider range of patients than the NIH criteria and are narrower than the Rotterdam criteria.

The clinical manifestations of PCOS are complex, and there is no single test that can diagnose PCOS. Diagnosis of PCOS could sometimes be difficult. Surveys have shown that there is worldwide dissatisfaction regarding the diagnosis and treatment experience in patients with PCOS, and one of the most serious problems is delayed diagnosis[6–8]. Delayed diagnosis could be caused by many factors, such as the complexity of PCOS manifestation and lack of unified diagnostic criteria, of which the most important and changeable factor was physicians’ knowledge of PCOS diagnostic criteria, especially ObGyn who took the most part in PCOS caring. Currently, there are no relevant survey data regarding ObGyn’s knowledge of PCOS diagnostic criteria in China. Although China’s medical services have been advancing rapidly in the past several decades, the development of the reproductive endocrinology subspecialty is still in its infancy. There are no subspecialties of reproductive endocrinology in most hospitals across China, and most of the ObGyn in China are required to diagnose and treat various gynaecological diseases, including PCOS. Whether ObGyn in China can accurately diagnose PCOS is a very important question.

Therefore, we designed a questionnaire to investigate the diagnostic criteria adopted by the Chinese ObGyn (including general obstetricians and gynaecologists [Ge-ObGyn] and reproductive endocrinologists [Re-ObGyn]) in the diagnosis of PCOS and whether the criteria were used correctly in their clinical practice.

## Methods

### Study design

We conducted an online survey via the largest nationwide continuing education platform of obstetrics and gynaecology across China from September 2019 to November 2019. The link of the questionnaire was sent to the WeChat groups of the education platform. Physicians were encouraged to take part in the survey in reward of free online courses.

### Participants

Physicians practising obstetrics and gynaecology across China were included in the study. The inclusion criteria were obstetricians and gynaecologists involved in the care of patients with PCOS, and at least one patient was cared for within the past year. The exclusion criterion was the incomplete content of the questionnaire submitted.

### Questionnaire development

We reviewed the literature regarding physicians’ diagnosis of PCOS and developed our questionnaire. The contents of the questionnaire were reviewed and revised by four specialists of reproductive endocrinology. We then tested the questionnaire by asking 30 obstetricians and gynaecologists to fill it out, and there was no ambiguity or doubt about the questionnaire contents.

### Questionnaire contents

Basic demographic data: The basic demographic data included gender, age, years in post, specialty, hospital classification, years involved in PCOS treatment, and the number of patients with PCOS treated annually.

Diagnostic criteria used: The diagnostic criteria used to diagnose PCOS in clinical practice and the correct use of the diagnostic criteria. We mainly investigated the use of the three most frequently used diagnostic criteria, namely, Rotterdam criteria, NIH criteria, and AE-PCOS criteria. First, we asked about the criteria they chose to diagnose PCOS. We then analysed whether they used the criteria correctly in clinical practice. The analysis method was based on whether they frequently used the items contained in the criteria they chose. The items included oligo-ovulation/anovulation, clinical hyperandrogenism, biochemical hyperandrogenism, and PCO. For those who chose the Rotterdam and AE-PCOS criteria, if the aforementioned four diagnostic items are all in ‘frequent’ application, then the respondent was considered to be the correct user of the diagnostic criteria. For those who chose the NIH criteria, when all the diagnostic items except for PCO are all in ‘frequent’ application, the respondent was considered to have used the criteria correctly.

The investigation was reviewed and approved by the Ethics Review Committee of the Peking Union Medical College Hospital, Chinese Academy of Medical Sciences at 25, June 2019 (number ZS2032). Participants were informed about the purpose of the study at the beginning of the questionnaire and the questionnaire was anonymous. Those who completed the questionnaire were deemed to have agreed to participate in the study.

### Analysis

Categorical variables were presented as frequencies and percentages. A multivariate logistic regression method was used to analyse the factors that influence the selection of PCOS diagnostic criteria and their correct application. The multivariates included gender, age, hospital classification, specialty, years in post, years involved in PCOS treatment, and the number of patients with PCOS treated annually. A P value of < 0.05 (two-tailed) was considered statistically significant. Data were analysed using SPSS software version 19.0 (IBM Inc., Somers, NY, USA).

## Results

### Characteristics of the respondents

A total of 3,213 responses were received, and 2,328 respondents met the inclusion criteria and were included in the final analysis. Of these, 96.6 % were women, 86.5 % worked in secondary or tertiary hospitals, 94.5 % were Ge-ObGyn, 73.5 % were 36–55 years old, and 73.8 % were more than 10 years in post. The vast majority (95.2 %) of the respondents had more than 1 year of experience in PCOS treatment, and 53.5 % had more than 5 years of experience. Moreover, 69.5 % saw less than 50 patients with PCOS annually (Table 1).

**Table 1:** Demographic characteristics of the respondents (*N*=2328)

	Overall (%) n (%)	Ge-ObGyn 2201 (94.5%)	Re-ObGyn 127 (5.5%)
**Gender**			
Female	2249 (96.6)	2130 (96.8)	119 (93.7)
Male	79 (3.4)	71 (3.2)	9 (6.3)
**Age (year)**			
18-25	26 (1.1)	22 (1.0)	4 (3.1)
26-35	504 (21.6)	462 (21.0)	42 (33.1)
36-45	992 (42.6)	955 (43.4)	37 (29.1)
46-55	719 (30.9)	678 (30.8)	41 (32.3)
≥56	87 (3.7)	84 (3.8)	3 (2.4)
**Hospital classification**			
Tertiary	951 (40.9)	855 (38.8)	96 (75.6)
Secondary	1062 (45.6)	1043 (47.4)	19 (15.0)
Primary	150 (6.4)	148 (6.7)	10 (7.9)
Others	165 (7.1)	155 (7.0)	2 (1.6)
**Years in post (y)**			
≤5	229 (9.8)	199 (9.0)	30 (23.6)
6–10	381 (16.4)	359 (16.3)	22 (17.3)
11–20	727 (31.2)	695 (31.6)	32 (25.2)
>20	991 (42.6)	948 (43.1)	43 (33.9)
**Years of PCOS caring (y)**			
<1	112 (4.8)	109 (5.0)	3 (2.4)
1–5	970 (41.7)	921 (41.8)	49 (38.6)
6–10	685 (29.4)	645 (29.3)	40 (31.5)
11–20	415 (17.8)	386 (17.5)	29 (22.8)
>20	146 (6.3)	140 (6.4)	6 (4.7)
**Number of patients with PCOS treated annually**			
**1-50**	1617 (69.5)	1582 (71.9)	35 (27.6)
**50-200**	535 (23.0)	479 (21.8)	56 (44.1)
**>200**	176 (7.6)	140 (6.4)	36 (28.3)

Data are presented as n (%)

### Diagnostic criteria used for PCOS

Overall, 48.2 % of the respondents used the AE-PCOS criteria, and 35.7 % used the Rotterdam criteria. Re-ObGyn (59.1 %), respondents who saw more than 200 patients with PCOS annually (60.2 %), and those who came from tertiary hospitals (46.6 %) were more likely to use the Rotterdam criteria. On the other hand, Ge-ObGyn, respondents who had less than 5 years of experience in PCOS treatment (53.5 %), those saw less than 50 patients annually (53.1 %), and those who came from non-tertiary hospitals (55.4 %) were more likely to choose the AE-PCOS criteria (Table 2).

**Table 2:** Diagnostic criteria for PCOS used and their associated physician characteristics using the multivariable logistic regression analysis (*N*=2328)

	Rotterdam criteria 832 (35.7) (ref.)	AE-PCOS criteria 1123 (48.2)		NIH criteria 281 (12.1)		Other criteria 43 (1.8)		Don’t know 49 (2.1)	
	n (%)	n (%)	Adjusted OR (95%CI)	n (%)	Adjusted OR (95%CI)	n (%)	Adjusted OR (95%CI)	n (%)	Adjusted OR (95%CI)
**Gender**									
Female (ref.)	799 (35.5)	1091 (48.5)	1.000	272 (12.1)	1.000	41 (1.8)	1.000	46 (2.0)	1.000
male	33 (41.8)	32 (40.5)	0.824 (0.490,1.387)	9 (11.4)	0.707 (0.327,1.530)	2 (2.5)	1.450 (0.320,6.567)	3 (3.8)	2.100 (0.559,7.884)
**Age (years)**									
18–25	10 (38.5)	12 (46.2)	0.964 (0.383,2.425)	2 (7.7)	0.340 (0.070,1.650)	0 (0)	-	2 (7.7)	3.039 (0.473,19.5080
26–35 (ref.)	159 (31.5)	244 (48.4)	1.000	79 (15.7)	1.000	10 (2.0)	1.000	12 (2.4)	1.000
36–45	343 (34.6)	513 (51.7)	1.050 (0.723,1.526)	100 (10.1)	0.828 (0.481,1.423)	18 (1.8)	1.024 (0.315,3.335)	18 (1.8)	2.334 (0.890,6.119)
46–55	282 (39.2)	317 (44.1)		91 (12.7)	1.378 (0.689,2.754)	13 (1.8)	0.703 (0.159,3.102)	16 (2.2)	5.385 (0.341,21.631)
≥56	38 (43.7)	37 (42.5)	1.118 (0.700,1.783) 1.024 (0.538,1.949)	9 (10.3)	1.048 (0.394,2.792)	2 (2.3)	0.774 (0.105,5.701)	1 (1.1)	2.952 (0.286,30.497)
**Hospital classification**									
Tertiary (ref.)	443 (46.6)	360 (37.9)	1.000	120 (12.6)	1.000	17 (1.8)	1.000	11 (1.2)	1.000
Secondary	310 (29.2)	577 (54.3)^*^	2.012 (1.627,2.488)	132 (12.4)^*^	1.557 (1.141,2.124)	17 (1.6)	1.300 (0.625,2.705)	26 (2.4)^*^	3.203 (1.442,7.115)
Primary	26 (17.3)	97 (64.7)^*^	3.724 (2.331,5.950)	15 (10.0)	1.962 (0.989,3.891)	5 (3.3)^*^	4.194 (1.360,12.932)	7 (4.7)^*^	7.959 (2.593,24.426)
Others	53 (32.1)	89 (53.9)^*^	1.820 (1.240,2.671)	14 (8.5)	0.917 (0.495,1.736)	4 (2.4)	1.753 (0.549,5.602)	5 (3.0)	2.777 (0.853,9.042)
**Specialty**									
Ge-ObGyn (ref.)	757 (34.4)	1090 (49.5)	1.000	264 (12.0)	1.000	41 (1.9)	1.000	49 (2.2)	1.000
Re-ObGyn	75 (59.1)	33 (26.0)^*^	0.513 (0.328,0.802)	17 (13.4)	0.833 (0.465,1.493)	2 (1.6)	0.696 (0.155,3.132)	0 (0)	-
**Years in post (y)**									
≤5 (ref.)	74 (32.3)	100 (43.7)	1.000	43 (18.8)	1.000	4 (1.7)	1.000	8 (3.5)	1.000
6–10	116 (30.4)	196 (51.4)	1.171 (0.763,1.797)	46 (12.1)	0.676 (0.386,1.183)	8 (2.1)	1.346 (0.360,5.035)	15 (3.9)	0.246 (0.059,1.032)
11–20	243 (33.4)	387 (53.2)	1.194 (0.718,1.985)	80 (11.0)	0.680 (0.339,1.364)	10 (1.4)	0.971 (0.191,4.930)	7 (1.0)	1.448 (0.473,4.433)
>20	399 (40.3)	440 (44.4)	0.733 (0.416,1.292)	112 (11.3)^*^	0.405 (0.182,0.902)	21 (2.1)	1.378 (0.238,7.960)	19 (1.9)	0.252 (0.053,1.193)
**Years of PCOS caring (y)**									
<1 (ref.)	19 (17.0)	58 (51.8)	1.000	20 (17.9)	1.000	3 (2.7)	1.000	12 (10.7)	1.000
1–5	290 (29.9)	521 (53.7)	0.617 (0.354,1.077)	119 (12.3)^*^	0.446 (0.225,0.884)	19 (2.0)	0.436 (0.113,1.677)	21 (2.2)^*^	0.144 (0.058,0.357)
6–10	288 (42.0)	293 (42.8)^*^	0.430 (0.239,0.773)	84 (12.3)^*^	0.412 (0.197,0.864)	9 (1.3)	0.230 (0.051,1.044)	11 (1.6)^*^	0.113 (0.038,0.336)
11–20	180 (43.4)	184 (44.3)	0.554 (0.299,1.026)	39 (9.4)^*^	0.349 (0.156,0.781)	7 (1.7)	0.324 (0.064,1.635)	5 (1.2)^*^	0.096 (0.025,0.362)
>20	55 (37.7)	67 (45.9)	0.747 (0.372,1.503)	19 (13.0)	0.580 (0.232,1.451)	5 (3.4)	0.757 (0.131,4.367)	0 (0.0)	-
**Number of patients with PCOS treated annually**									
1-50 (ref.)	482 (29.8)	859 (53.1)	1.000	2 (12.5)	1.000	30 (1.9)	1.000	44 (2.7)	1.000
50-200	244 (45.6)	215 (40.2)^*^	0.417 (0.282,0.616)	62 (11.6)	0.755 (0.530,1.076)	10 (1.9)	0.894 (0.401,1.995)	4 (0.7)	0.407 (0.137,1.204)
>200	106 (60.2)	49 (27.8)^*^	0.673 (0.531,0.854)	17 (9.7)^*^	0.535 (0.298,0.961)	3 (1.7)	0.625 (0.167,2.338)	1 (0.6)	0.395 (0.050,3.127)

^*^P<0.05, multivariable logistic regression analysis. *OR* odds ratio. The Rotterdam criteria was the reference category of dependent variables. ref., reference category.

### Correct application rate of the diagnostic criteria for PCOS

We also investigated the application of the four major diagnostic items involved in the three most frequently used international diagnostic criteria, of which oligo-menstruation was the most frequently used diagnostic item (74.1 % of the total responders), followed by biochemical hyperandrogenism (70.0 %), clinical hyperandrogenism (66.1 %), and PCO (64.0 %) (Table 3).

**Table 3:** Frequency in the use of criteria items to diagnose PCOS (*N*=2328)

	Overall (*N*=2328)	Rotterdam criteria (*n*=832)	AE-PCOS criteria (*n*=1123)	NIH criteria (*n*=281)	Other criteria (*n*=43)	Do not know (*n*=49)
Oligo-menstruation						
Never	79 (3.4)	21 (2.5)	44 (3.9)	10 (3.6)	2 (4.7)	2 (4.1)
Rarely	173 (7.4)	27 (3.2)	109 (9.7)	23 (8.2)	3 (7.0)	11 (22.4)
Sometimes	352 (15.1)	62 (7.5)	218 (19.4)	49 (17.4)	10 (23.3)	13 (26.5)
Often	17243 (74.1)	722 (86.8)	752 (67.0)	199 (70.8)	28 (65.1)	23 (46.9)
Clinical hyperandrogenism						
Never	72 (3.1)	23 (2.8)	38 (3.4)	8 (2.8)	1 (2.3)	2 (4.1)
Rarely	201 (8.6)	48 (5.8)	109 (9.7)	33 (11.7)	3 (7.0)	8 (16.3)
Sometimes	517 (22.2)	151 (18.1)	265 (23.6)	75 (26.7)	15 (34.9)	11 (22.4)
Often	1538 (66.1)	610 (73.3)	711 (63.3)	165 (58.7)	24 (55.8)	28 (57.1)
Biochemical hyperandrogenism						
Never	58 (2.5)	14 (1.7)	30 (2.7)	11 (3.9)	1 (2.3)	2 (4.1)
Rarely	185 (7.9)	49 (5.9)	85 (7.6)	35 (12.5)	5 (11.6)	11 (22.4)
Sometimes	455 (19.5)	154 (18.5)	226 (20.1)	53 (18.9)	12 (27.9)	10 (20.4)
Often	1630 (70.0)	615 (73.9)	782 (69.6)	182 (64.8)	25 (58.1)	26 (53.1)
PCO						
Never	102 (4.4)	36 (4.3)	52 (4.6)	10 (3.6)	3 (7.0)	1 (2.0)
Rarely	235 (10.1)	83 (10.0)	117 (10.4)	27 (9.6)	3 (7.0)	5 (10.2)
Sometimes	500 (21.5)	176 (21.2)	243 (21.6)	62 (22.1)	11 (25.6)	8 (16.3)
Often	1491 (64.0)	537 (64.5)	711 (63.3)	182 (64.8)	26 (60.5)	35 (71.4)
Correct use of diagnostic criteria^*^	729 (31.3)	343 (41.2)	361 (32.1)	25 (8.9)	-	-

Data are presented as n (%)

**Fig. 1:**
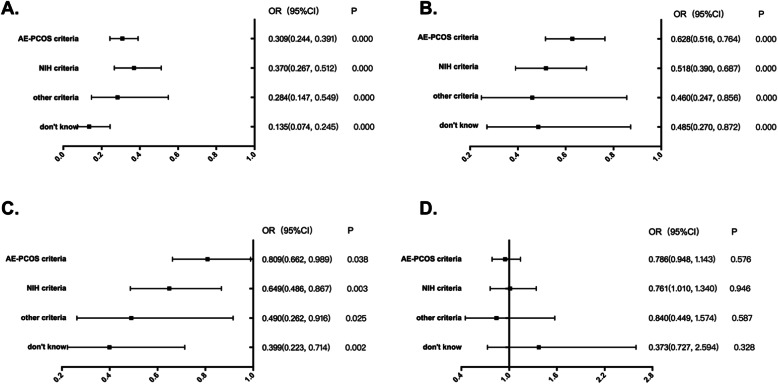
Association between the ‘frequent’ application rates of oligo-menstruation (**a**), clinical hyperandrogenism (**b**), biochemical hyperandrogenism (**c**), and polycystic ovary (PCO) (**d**) and diagnostic criteria used OR, odds ratio; binary logistic regression analysis. The Rotterdam criteria are the reference category of independent variables

The respondents who used the Rotterdam criteria were more likely to frequently use oligomenorrhoea (86.8 %), biochemical hyperandrogenism (73.9 %), and clinical hyperandrogenism (73.3 %) as diagnostic items than those who used any of the other diagnostic criteria. No significant difference was noted in the frequent use of PCO as a diagnostic item among respondents who chose different diagnostic criteria (Table 3; Fig. 1).

Only 31.3 % of the respondents correctly applied the diagnostic criteria they used. The correct application rate was the highest among the respondents who used the Rotterdam criteria (41.4 %), it was 32.1 % in those who used the AE-PCOS criteria, and it was the lowest in those who used the NIH criteria (8.9 %) (Table 3).

Compared with Ge-ObGyn, respondents who had less than 1 year of experience in PCOS treatment, and those who saw less than 50 patients with PCOS annually, Re-ObGyn (odds ratio [OR], 1.492; 95 % CI, 1.014–2.196; *P* = 0.043), respondents who had more than 1 year of experience in PCOS treatment (OR value range of each age range, 1.788–2.574; *P* < 0.05), and those who saw more than 200 patients with PCOS annually (OR, 1.639; 95 % CI, 1.157–2.323; *P* = 0.005) were more likely to use the diagnostic criteria correctly. Compared with tertiary hospitals, the diagnostic accuracy of non-tertiary hospitals was lower (OR, 0.649; 95 % CI, 0.435–0.968; *P* = 0.034) (Table 4).

**Table 4:** Association between the correct use of diagnostic criteria and physicians’ characteristics (*N*=2328)

	n (%) 729 (31.3%)	P	Adjusted OR	95% CI
**Gender**				
Female (ref.)	703 (31.3)		1.000	
Male	26 (32.9)	0.993	0.998	0.611, 1.630
**Age (years)**				
18–25	12 (46.2)	0.076	2.130	0.923, 4.193
26–35 (ref.)	149 (29.2)		1.000	
36–45	315 (31.8)	0.667	1.079	0.763, 1.525
46–55	223 (31.0)	0.803	1.058	0.681, 1.643
≥56	30 (34.5)	0.660	1.146	0.623, 2.108
**Hospital classification**				
Tertiary (ref.)	323 (34.0)		1.000	
Secondary	329 (31.0)	0.921	1.010	0.825, 1.237
Primary	40 (26.7)	0.439	0.854	0.573, 1.274
Others	37 (22.4)	0.034^*^	0.649	0.435, 0.968
**Specialty**				
Ge-ObGyn (ref.)	673 (30.6)		1.000	
Re-ObGyn	56 (44.1)	0.043^*^	1.492	1.014, 2.196
**Years in post (y)**				
≤5 (ref.)	71 (31.0)		1.000	
6–10	114 (29.9)	0.854	0.963	0.648, 1.432
11–20	239 (32.9)	0.874	0.963	0.602, 1.540
>20	305 (30.8)	0.315	0.762	0.449, 1.295
**Years of PCOS caring (y)**				
<1 (ref.)	21 (18.8)		1.000	
1–5	293 (30.2)	0.021^*^	1.816	1.093, 3.018
6–10	206 (30.1)	0.035^*^	1.788	1.041, 3.072
11–20	156 (37.6)	0.001^*^	2.558	1.450, 4.510
>20	53 (36.3)	0.004^*^	2.574	1.353, 4.899
**Number of patients with PCOS treated annually**				
1-50 (ref.)	469 (29.0)		1.000	
50-200	180 (33.6)	0.344	1.117	0.888, 1.404
>200	80 (45.5)	0.005^*^	1.639	1.157, 2.323

Data are presented as n (%)

^*^P<0.05, multivariate binary logistic regression model. *OR* odds ratio. *Ref* reference category

## Discussion

### Main findings

To the best of our knowledge, this is the first survey to evaluate the diagnostic criteria used for PCOS among ObGyn across China. We found that the AE-PCOS criteria were the most frequently used criteria (48.2 %), followed by the Rotterdam criteria (35.7 %) and NIH criteria (12.1 %). The accurate application rate of these criteria was quite low (31.3 %). Specialty, PCOS-related work experience, and hospital classification were the relevant impact factors.

### Strengths and limitations

This was the largest survey regarding physicians’ diagnosis of PCOS worldwide. In a similar international survey, only 1,495 physicians were included. In our study, data were collected in a much shorter period of time, which could minimise the influence of time.

The main limitation was that there may be a selection bias. First, the response rate of the survey was not high. Currently, there are approximately 100,000 obstetricians and gynaecologists across China, and only 2,000 ObGyn participated in the survey. Therefore, there may be concerns that they may not accurately reflect the overall level. Second, this research was initiated via the largest continuing education and training platform of obstetrics and gynaecology. We often organise gynaecological endocrine knowledge training courses on the platform. The knowledge level of ObGyn in this survey regarding PCOS diagnosis may be higher than the overall level across China. However, this will not change our conclusion. The survey showed that most ObGyn could not accurately use the diagnostic criteria for PCOS, so the overall level may be even worse.

### Interpretation

The use of diagnostic criteria for PCOS varies in different regions across the world, but the most frequently used criteria are still the Rotterdam criteria[9–11]. An international online survey showed that 67 % of physicians used the Rotterdam criteria for PCOS; more than three-fourths 3/4 (77.5 %) of physicians in Europe, approximately half (49.8 %) in North America, and most (80.2 %) in Asia and other regions used the Rotterdam criteria, and only 7.2 % used the AE-PCOS criteria[9]. In another report, 67 % of physicians across North Europe used the Rotterdam criteria, and differences existed among countries, with the lowest in Estonia (43 %) and highest in Iceland (93 %)[10] In another international online survey, 82 % of physicians used the Rotterdam criteria, with 100 % in Australia, 81 % in Asia, and 70 % in the United States[11]. In these surveys, the proportion of using the other two diagnostic criteria was no more than 12 %, and the reported ranges were 2.5–12 % and 1–11.8 % for the NIH and AE-PCOS criteria, respectively[9–11]. The proportions of physicians who did not know the diagnostic criteria or who used other criteria were approximately 18–26 %[9–11]. From these reports, we can see that the use of diagnostic criteria for PCOS varies widely among different regions across the world, but the most frequently used criteria were the Rotterdam criteria. In this survey, the AE-PCOS criteria were the most frequently used (48.2 %), followed by the Rotterdam criteria (35.7 %), which is quite different from the data reported abroad and also very different from our experience. In our training courses and clinical work, the Rotterdam criteria are much more frequently used than the AE-PCOS criteria. The reasons why they choose the AE-PCOS criteria in the questionnaire may be that they actually do not know the actual name of the diagnostic criteria or do not know how to diagnose PCOS and they choose the AE-PCOS criteria just because the name contains the word ‘PCOS’.

Although the Rotterdam criteria are the most commonly used criteria worldwide, it has been increasingly recognised that race and ethnic differences could influence the clinical presentations and phenotypes of PCOS[12–15]. Asians are less hirsute[16] and have a higher prevalence of polycystic ovarian morphology[17], and the cutoff of the hirsutism score needs to be lower than that of Caucasians[18, 19]. Genetic background plays important roles in PCOS ethnic variations[20, 21]. Several genetic factors may modify the clinical manifestations of PCOS in different ethnic groups[22], and informative gene loci associated with PCOS have been reported to be different between Asian and European patients[21]. Some countries have made their own diagnostic criteria based on the usual clinical presentation of PCOS[23, 24]. However, race and ethnicity have not yet been considered in these three most commonly used international diagnostic criteria for PCOS[25]. Further studies are needed to take race and ethnicity into account to assess the appropriateness of the use and interpretation of different diagnostic criteria for PCOS.

Further analysis found that the respondents who chose the Rotterdam criteria were more likely to accurately use the diagnostic criteria than those who chose the AE-PCOS criteria, indicating that it is very likely that some respondents were not clear about the diagnostic criteria and they chose the AE-PCOS criteria just because the name contains the word ‘PCOS’, in which they guessed that this might be the right one. Among the respondents who used the NIH criteria, 98 (34.9 %) selected the four diagnostic indicators as ‘frequent’, indicating that there were also some respondents who were familiar with the diagnostic items but not clear about the name of the diagnostic criteria. The correct application rate of the NIH criteria was 8.9 %. In general, the proportion of the correct use of diagnostic criteria for PCOS was very low (less than one-third), indicating that most ObGyn did not have sufficient knowledge of the diagnostic criteria commonly used for PCOS.

Re-ObGyn, respondents who had more than 1 year of experience in PCOS, those who saw more than 200 patients with PCOS annually, or those who came from tertiary hospitals were more likely to use the diagnostic criteria for PCOS correctly. PCOS is one of the most common diseases in the clinical practice of Re-ObGyn. Re-ObGyn had more years of experience and were more professional in PCOS treatment than Ge-ObGyn. The correct application rate of diagnostic criteria was related to PCOS-related work experience, indicating that professionalism is an important factor affecting the correct application of diagnostic criteria. This is consistent with previous investigations in the United States, which showed that 27 % of doctors did not know the diagnostic criteria for PCOS, and comparing them with reproductive physicians, the proportion of obstetrics and gynaecology who did not know the diagnostic criteria for PCOS was higher (37.1 % vs. 5.9 %, *P* < 0.05)[26]. Therefore, it is necessary to strengthen the construction of PCOS-related subspecialties. In tertiary hospitals, continuing education resources are more abundant, physicians have a higher academic level and more opportunities to participate in academic trainings or meetings, and medical service quality is higher[27],[28]. Therefore, it is expected that physicians from tertiary hospitals performed better in the use of diagnostic criteria for PCOS.

Physicians’ knowledge of PCOS has a great impact on patients’ experience of PCOS diagnosis and treatment. Previous surveys have shown that there is worldwide dissatisfaction with the diagnosis and treatment experience of patients with PCOS[8, 29–31]. One of the biggest problems was delayed diagnosis. An international survey showed that 33.6 % of patients with PCOS were not diagnosed until more than 2 years of hospital visits, 47.1 % were required to see more than two physicians before diagnosis was made, and 42.4 % were not satisfied with the diagnosis experience[6]. An Australian survey showed that 46 % of patients with PCOS were diagnosed after 6 months from the initial hospital visit, one-fourth were diagnosed after 2 years, and 39 % needed to see more than two physicians before diagnosis was made[8]. A British survey showed that the median time for PCOS diagnosis was 6–12 months[32]. There are many reasons for delays in diagnosis. These are related to the following: there is the lack of a single diagnostic test for PCOS, the criteria are not unified, the primary medical institution may not provide ultrasound examination, and there is the lack of a multidisciplinary joint management model[33]. Unfamiliar with the characteristics of and diagnostic criteria for PCOS and the lack of corresponding continuing education resources[8] are the main factors that physicians are unfamiliar with the knowledge they should have known. Trainings and the construction of specialties are also very important contributing factors[34]. A survey in the United States showed that the satisfaction of patients with PCOS with specialists was not reduced compared with that of non-PCOS patients, while their satisfaction with primary physicians was significantly reduced[31]. Therefore, there is an international call for continuing education and resource support for PCOS related to physicians to improve their understanding of PCOS features and the diagnosis and treatment experience of patients with PCOS and provide prompt timely diagnosis and treatment[6, 35, 36].

## Conclusions

Our investigation found that only one-third of obstetricians and gynaecologists correctly used the PCOS diagnostic criteria across China, in which the proportion was very low. Specialty, PCOS-related work experience, and hospital classification are the relevant impact factors. There is an urgent need to strengthen the continuing education of knowledge regarding PCOS diagnosis among obstetricians and gynaecologists in China and the construction of gynaecological endocrinology subspecialty and its personnel training to improve the medical service quality of PCOS.

The manuscript has been read and approved by all the authors. All the authors have met the requirements for authorship. All the authors believe that the manuscript represents honest work.

## Data Availability

Data and materials are summarized in the manuscript, figures, and tables.
